# How scientists and institutions should respond

**DOI:** 10.7554/eLife.106702

**Published:** 2025-03-25

**Authors:** Ran Blekhman

**Affiliations:** 1 Section of Genetic Medicine, Department of Medicine, University of Chicago Chicago United States

**Keywords:** Science Under Threat in the United States, science, politics, science funding, basic research, careers in science, early-career researchers, None

## Abstract

Individual researchers and university leaders need to make the case for science to their elected representatives and to the public at large.

The new Trump administration has wasted no time launching a multi-pronged assault on the scientific enterprise in the United States. Mass firings at the National Institutes of Health (NIH) and the National Science Foundation have gutted these agencies of institutional knowledge and expertise. The slashing of indirect costs is a devastating financial blow that will affect research universities in every state and undermine the infrastructure that makes research possible: some states could lose hundreds of millions of dollars if this policy (which is being challenged in the courts) is not reversed. Meanwhile, grant funding and reviews are facing significant delays, with some active grants being terminated without notice, leaving scientists in limbo and critical research stalled.

Make no mistake: this is not just bad for science – it is bad for America. Our global leadership in biomedicine, technology and basic science discovery has driven our economic growth and improved countless lives. The impact is staggering: over 99% of newly approved drugs were developed with contributions from NIH-funded research. For example, the development of Trikafta – a revolutionary treatment for cystic fibrosis that has transformed a fatal disease into a manageable condition for thousands of Americans – relied on basic research funded by the NIH. The shortsightedness of these cuts betrays a fundamental misunderstanding of how scientific progress works. Innovation does not happen in fits and starts based on political whims; it requires sustained, stable investment. When we undermine our scientific infrastructure, we are not just losing current research, we are compromising our future competitive edge and potentially driving a generation of talent away from scientific careers or toward more science-friendly countries.

Individual scientists cannot afford to retreat to the lab and hope this storm passes. Instead, we can take concrete action. First, we must contact our representatives – repeatedly. Congressional offices track communications from constituents, and the volume of phone calls and emails matters. And there are other actions we can take. Join the advocacy efforts of scientific societies, which often have established channels to policymakers. Organize letter-writing campaigns and meetings with congressional staffers, where we can share concrete examples of how funding cuts will impact our local community: the jobs that will be lost, the patients who will no longer benefit from medical research, and the students whose careers will be put at risk. Participate in public demonstrations for science, such as the Stand Up For Science rallies – our physical presence at these events matters and helps showcase that scientists are engaged citizens. Stay informed about policy developments via trusted news sources so we can act strategically, but avoid getting consumed by the daily news cycle and constant updates that can derail our research focus. Make our research accessible to the public through community events, social media, and local news. The more voters understand what’s at stake, the more pressure will build on elected officials. For principal investigators, maintain transparent communication with your lab members about the funding situation and contingency plans – students and postdocs deserve honesty about how these changes might affect their careers. And critically, we should support our colleagues, especially those early in their careers who face the most precarious situations. Share resources, collaborate strategically, and mentor those navigating these troubled waters.

As for institutions, several universities have taken meaningful first steps by joining lawsuits that have temporarily halted some of the administration’s most damaging actions. This legal resistance is crucial. However, many of the same institutions have also implemented cost-cutting measures, such as hiring freezes and, in some cases, freezes on the admission of new PhD students. While these defensive measures may seem prudent, they actually compound the damage by further constricting the research pipeline, and institutions should consider more nuanced approaches. PhD students are the lifeblood of scientific progress and discovery, so rather than freezing all admissions, it would be better to pause admissions selectively for programs with funding gaps while maintaining them in areas with stronger funding prospects. Other approaches could include: adjusting cohort sizes based on each program’s specific circumstances, rather than making uniform cuts; developing more flexible funding packages that incorporate additional teaching assistantships; and creating more industry partnerships that can subsidize graduate training while providing students with exposure to other careers.

University leaders also need to move beyond reactive budget cuts and toward proactive solutions. Public universities should leverage their relationships with state legislators to advocate for stopgap funding. Universities with substantial endowments should create emergency bridge funding for at-risk research programs, particularly those that lack the reserves to sustain them through prolonged funding interruptions. Institutions can collaborate to identify cost-saving measures that protect research capacity, including resource-sharing initiatives and public–private partnerships. University leaders must also step up their outreach to the general public, clearly communicating how research funding translates to economic growth, medical breakthroughs, and community benefits that extend far beyond campus boundaries.

Critically, universities must maintain transparent, frequent communication with their faculty and scientists: holding regular town halls to provide updates on the situation, clearly articulating their advocacy efforts, and being forthright about their plans and the financial outlook. This should include specific guidance for faculty on navigating grant applications during this period, strategies for sustaining research programs, and information on the institutional resources available to support them. Uncertainty breeds anxiety, so regular, honest communication from leadership will demonstrate respect for those whose careers hang in the balance. The legal challenges buy us time, but we need comprehensive, proactive strategies to sustain scientific infrastructure.

We have weathered political attacks on science before, but the scale and speed of these changes demand unprecedented mobilization. By working collectively – scientists raising our voices, institutions leveraging their influence, and the public recognizing what’s at stake – we can protect the scientific enterprise that drives American innovation.

